# Usefulness of *Opuntia* spp. on the Management of Obesity and Its Metabolic Co-Morbidities

**DOI:** 10.3390/nu16091282

**Published:** 2024-04-25

**Authors:** Iker Gómez-García, Alfredo Fernández-Quintela, Marcela González, Saioa Gómez-Zorita, Begoña Muguerza, Jenifer Trepiana, María P. Portillo

**Affiliations:** 1 Nutrition and Obesity Group, Department of Nutrition and Food Science, University of the Basque Country (UPV/EHU) and Lucio Lascaray Research Institute, 01006 Vitoria-Gasteiz, Spain; iker.gomez@ehu.eus (I.G.-G.); alfredo.fernandez@ehu.eus (A.F.-Q.); saioa.gomez@ehu.eus (S.G.-Z.); mariapuy.portillo@ehu.eus (M.P.P.); 2 Bioaraba Health Research Institute, 01006 Vitoria-Gasteiz, Spain; 3 CIBERobn Physiopathology of Obesity and Nutrition, Institute of Health Carlos III, 28029 Madrid, Spain; 4 Nutrition and Food Science Department, Faculty of Biochemistry and Biological Sciences, National University of Litoral and National Scientific and Technical Research Council (CONICET), Santa Fe 3000, Argentina; maidagon@fbcb.unl.edu.ar; 5 Nutrigenomics Research Group, Departament de Bioquímica i Biotecnología, Universitat Rovira i Virgili, 43007 Tarragona, Spain; begona.muguerza@urv.cat

**Keywords:** *Opuntia* spp., nopal, prickly pear, *Opuntia ficus-indica*, obesity, metabolic co-morbidities

## Abstract

The plants of the *Opuntia* genus mainly grow in arid and semi-arid climates. Although the highest variety of wild species is found in Mexico, *Opuntia* spp. is widely distributed throughout the world. Extracts of these cacti have been described as important sources of bioactive substances that can have beneficial properties for the prevention and treatment of certain metabolic disorders. The objective of this review is to summarise the presently available knowledge regarding *Opuntia ficus-indica* (nopal or prickly pear), and some other species (*O. streptacantha* and *O. robusta*) on obesity and several metabolic complications. Current data show that *Opuntia ficus-indica* products used in preclinical studies have a significant capacity to prevent, at least partially, obesity and certain derived co-morbidities. On this subject, the potential beneficial effects of *Opuntia* are related to a reduction in oxidative stress and inflammation markers. Nevertheless, clinical studies have evidenced that the effects are highly contingent upon the experimental design. Moreover, the bioactive compound composition of nopal extracts has not been reported. As a result, there is a lack of information to elucidate the mechanisms of action responsible for the observed effects. Accordingly, further studies are needed to demonstrate whether *Opuntia* products can represent an effective tool to prevent and/or manage body weight and some metabolic disorders.

## 1. Introduction

The genus *Opuntia* is characterised by its ability to thrive in challenging environmental conditions, given its capacity to grow in arid and semi-arid zones [1]. *Opuntia* plants, classified within the *Cactaceae* family, are notable for their globose stems adorned with thorns, referred to as cladodes. These cladodes are flat, oval, fleshy segments that bear spikes (called glochids). The genus *Opuntia* is further distinguished by the production of pear-shaped fruits exclusive to this genus, commonly known as prickly pears. To date, opuntioid cacti encompass over 250 species, predominantly distributed in the Americas, with additional occurrences in Asia, Africa, Oceania and certain regions of the Mediterranean area [2]. Mexico is recognised for hosting the most diverse array of species within this genus, reaching up to 126 different species of *Opuntia* [3].

*Opuntia* fruits are extensively consumed, and the products derived from the plant find applications in the pharmaceutical, food and cosmetic industries, rendering them of considerable economic significance [4]. *Opuntia* has been identified as a viable alternative for animal feeding due to its low water requirements and high productivity; therefore, *Opuntia* plantations have the potential to contribute to the development of agriculture [5]. Furthermore, *Opuntia* cultivations fulfil ecosystem protection functions by serving as habitats for a diverse range of living organisms, supplying raw material for soil development, offering protection against erosion and potentially participating in phytoremediation processes for contaminated water and soil [6].

For years, health benefits associated with the *Opuntia* genus have been documented in folk medicine, and this traditional knowledge remains highly relevant in many indigenous communities to this day [7]. From the scientific point of view, it has been observed that extracts of opuntioid cacti, obtained from fruits, cladodes or flowers, can exert beneficial properties for the prevention and treatment of certain disorders, such as obesity, type-2 diabetes, cardiovascular diseases, non-alcoholic fatty liver disease and several types of cancer [3,8,9,10,11,12].

The health-promoting properties of *Opuntia* are primarily attributable to its antioxidant and anti-inflammatory properties, mainly associated with the high content of bioactive compounds. These include phenolic compounds, betalains and phytosterols, as well as certain polysaccharides and vitamins [7,13,14,15]. The presence of phytochemicals such as phenolic acids, pigments or antioxidants have been found in all *Opuntia* products, including roots, cladodes, seeds, fruits or juice, although the amounts vary depending on the variety and the part of the plant [3]. The content of bioactive compounds is mainly influenced by the soil where the cactus is grown and thus by the geographical area of cultivation. Moreover, this chemical profile is highly variable and contingent on climatic conditions [16]. Therefore, these variations can affect the biological properties of each variety [17].

Taking all of the above into consideration, the aim of this review is to summarise the documented knowledge concerning the beneficial effects of *Opuntia* spp. on obesity co-morbidities. Presently, several investigations have been conducted both in *in vivo* models and in humans, *Opuntia ficus-indica* cactus (also known as nopal or prickly pear) emerging as the most extensively investigated species in the majority of these studies. Additionally, some research has been conducted with *Opuntia robusta* and *Opuntia streptacantha*.

## 2. Search Strategy

A bibliographic search was conducted to identify studies included in the PubMed medical database up to May 2023, using different combinations of the following keywords: obesity, obesity management, body weight, anti-obesity agents, weight loss, overweight, metabolic syndrome, *Opuntia* and cactus. Therefore, only original articles written in English were included. From the initially collected articles, a total of 19 studies were retained following the screening process, which involved evaluating the title, abstract and full text.

## 3. Effects of *Opuntia* spp. on Obesity-Related Co-Morbidities in Preclinical Studies

The most investigated cactus species in *in vivo* experimental models is *Opuntia ficus-indica*. In fact, there is only one study conducted on a different species, specifically the investigation reported by Héliès-Toussaint et al. (2020) focusing on *Opuntia streptacantha* [18]. These *in vivo* studies have been performed using either genetic obese animal models or diet-induced obesity models (Table 1). Morán-Ramos et al. (2012) [10] aimed at studying the effect of *Opuntia ficus-indica* cladodes in Zucker (*fa*/*fa*) rats, a model of genetic obesity. For this purpose, rats were divided into two groups and were fed for seven weeks with either a control diet or a diet supplemented with a dehydrated extract of *Opuntia ficus-indica* cladodes, collected in Mexico. Diets were supplemented with an amount of *Opuntia ficus-indica* sufficient to yield 4% of fibre, replacing the cellulose content present in the control diet. No changes were observed in weight gain, although the daily food intake was increased in the *Opuntia ficus-indica*-treated group (+2.1%, *p* < 0.01). These animals showed decreased serum cholesterol (−31%, *p* < 0.001), alanine aminotransferase (−44%, *p* < 0.05) (ALT) and aspartate aminotransferase (−30%, *p* < 0.01) (AST) levels, whereas serum adiponectin levels significantly increased in the supplemented group (+75%, *p* < 0.01).

A substantial body of research has also focused on the effects of cladode extracts from *Opuntia ficus-indica* in murine models (mouse or rat) characterised by obesity induced through high-fat feeding. These studies utilised diets supplying fat with the range of 30–60% of energy, or alternatively, a cafeteria diet. In this line, Aboura et al. (2019) [19] studied the effect of an aqueous extract of *Opuntia ficus-indica* cladodes on mice fed with a high-fat diet (60% of energy from fat), supplemented or not with 1% of the infusion of *Opuntia ficus-indica* cladodes (administered daily in the drinking water) for six weeks. The infusion contained 6.99 mg of polyphenols/100 mL. At the end of the experimental period, the mice fed with the high-fat diet (HFD) significantly increased both body and adipose tissue weight when compared with the mice fed with the standard diet (*p* < 0.05). The mice supplemented with the *Opuntia ficus-indica* infusion showed lower values for both parameters (*p* < 0.05), although they did not reach the values observed in the control mice. This suggests that the supplementation partially prevented obesity. The same results were found in triglyceride (TG) (−18%, *p* < 0.05), total cholesterol (−24%, *p* < 0.01), glucose (−32%, *p* < 0.05), insulin (−24%, *p* < 0.05), interleukin 6 (IL-6) (−33%, *p* < 0.05) and tumour necrosis factor α (TNFα) (−20%, *p* < 0.05) plasma levels.

Moreover, the authors measured the gene expression of leptin and pro-inflammatory cytokines (IL-1β, IL-6 and TNF-α, but not IL-10) in adipose tissue, noting that it was higher in mice fed with the obesogenic diet. Interestingly, the infusion of *Opuntia ficus-indica* totally prevented this effect (*p* < 0.05). In the case of adiponectin, despite the decrease in its gene expression induced by the high-fat diet, the infusion was not able to significantly prevent this effect. In summary, the administration of *Opuntia ficus-indica* cladodes as an infusion exhibits a protective effect against obesity and inflammation induced by a high-fat diet.

Sánchez-Tapia et al. (2017) [20] studied the potential of *Opuntia ficus-indica* supplementation to mitigate the metabolic repercussions of obesity by modifying the gut microbiota and preventing metabolic endotoxemia in rats subjected to a high-fat high-sucrose diet. During the first seven months of the experimental period, rats were fed with a standard diet or a high fat-sucrose diet (45% energy from fat added to the diet and 5% from sucrose added to the drinking water). Subsequent to this period, rats continued with their respective diets. However, the animals receiving the obesogenic diet were distributed into four groups for an additional month: rats fed with a standard diet, supplemented or not with dehydrated *Opuntia ficus-indica* cladodes that provided 5% of dietary fibre from nopal instead of cellulose, and rats on the same obesogenic diet, supplemented or not with the dehydrated *Opuntia ficus-indica* at the equivalent dose.

At the end of the experimental period, rats subjected to the obesogenic diet for eight months showed significantly higher body weight than the other groups (+23.6%). By contrast, rats from cohorts that received the standard diet throughout the entire experimental period, as well as rats from the group initially exposed to the obesogenic diet and subsequently transitioned to the standard diet supplemented with *Opuntia ficus-indica*, displayed the lowest values in this parameter. The remaining groups exhibited intermediate values, and it is important to note that these differences were not attributed to variations in food intake.

Rats fed the obesogenic diet during the whole treatment period showed higher glucose and insulin serum levels, although *Opuntia ficus-indica* supplementation prevented these increases. Moreover, this enriched diet completely adverted the boost in serum triglyceride (*p* = 0.0054), total-cholesterol (*p* < 0.0001), LDL-cholesterol (*p* < 0.0001) and leptin (*p* < 0.0001) increases induced by the high-fat feeding, although leptin was an exception. These results demonstrate that *Opuntia ficus-indica* was also effective in preventing obesity-related comorbidities.

To ascertain whether the effects of *Opuntia ficus-indica* were related to alterations in gut microbiota, an investigation into bacterial diversity was conducted. The control group exhibited the highest diversity, while the group of rats fed the obesogenic diet without supplementation throughout the entire experimental period displayed the lowest. The remaining groups demonstrated intermediate levels of diversity. At the phylum level, it was observed that the abundance of *Bacteroidetes* increased with respect to that of *Firmicutes* in the rats supplemented with *Opuntia ficus-indica*. At the genus level, the supplementation boosted *Anaeroplasma*, *Prevotella* and *Ruminucoccus* and reduced *Faecalibacterium*, *Clostridium* and *Butyricicoccus*.

In addition, the obesogenic diet led to a reduction in intestinal mucus layer thickness compared with the control group, and this effect was concomitant with a decrease in intestinal occludin-1 protein. *Opuntia ficus-indica* supplementation avoided these effects, suggesting an improvement in intestinal permeability. Moreover, *Opuntia ficus-indica* attenuated the elevation of serum lipopolysaccharide induced by the diet.

In assessing the expression of genes related to oxidative stress and inflammation in adipose tissue, the authors observed that *Opuntia ficus-indica* supplementation reduced the expression of leptin, NADPH oxidase (*Nox*), *Tnf-α* and amyloid precursor protein (*App*) genes. Furthermore, the expression of *Tnf-α* gene was lower than in the control group.

Héliès-Toussaint et al. (2020) [18] analysed the effects of a cladode extract *from Opuntia ficus-indica* on Sprague–Dawley rats fed with a high-fat diet (30% of energy from fat), supplemented or not with 0.5 *w*/*w* of the extract for eight weeks. At the end of the experimental period, increased final body weight was observed in both groups fed with a high-fat diet, compared to the control group. However, rats supplemented with *Opuntia ficus-indica* exhibited a significantly reduced body mass gain compared to rats subjected to the non-supplemented high-fat diet (−12.5%, *p* < 0.05). Additionally, supplementation with *Opuntia ficus-indica* led to a reduction in abdominal fat weight (−20%), serum glucose, insulin levels (−20%) and serum triglyceride levels compared to the group fed with the non-supplemented high-fat diet, although these changes did not reach statistical significance. The group supplemented with *Opuntia ficus-indica* showed an increased amount of triglycerides in faeces compared to the control group (+23%, *p* < 0.05). This finding may align with a reduction in body weight. High-fat feeding led to decreased adiponectin serum levels and increased leptin serum levels, effects that were significantly prevented by *Opuntia ficus-indica* supplementation (+26% of adiponectin levels and −30% of leptin levels).

Urquiza-Martínez et al. (2020) [21] studied the hypothalamic function through the introduction of *Opuntia ficus-indica* flour in either a standard diet or a high-fat diet. C57Bl/6J mice were initially stratified into two cohorts, with one group receiving a standard diet and the other a high-fat diet (60% of energy from fat) over a period of twelve weeks. Subsequently, each diet cohort was further divided into two subgroups: one supplemented with *Opuntia ficus-indica* cladodes flour (17% *w*/*w*) and the other enriched with fibre (an amount similar to that provided by the cactus flour), extending the dietary intervention for additional weeks.

The cactus flour had no effect on body weight when added to the standard diet. However, it normalised body weight when it was administered with the high-fat diet. This anti-obesity effect was due, at least in part, to the reduction observed in epididymal (−57.9%, *p* < 0.001) and retroperitoneal (−70.3%, *p* < 0.001) adipose tissues. In addition to its anti-obesity effect, *Opuntia-ficus indica* flour improved glucose metabolism control, as evidenced by the outcomes of the glucose tolerance test. *Opuntia ficus-indica* flour increased food intake when administered in conjunction with the standard diet. Conversely, its addition to the high-fat diet resulted in a reduction in this parameter. Therefore, the investigation focused on feeding behaviour, a pivotal aspect in the regulation of food intake. The integration of cactus flour with the standard diet delayed the point of satiation, while its combination with the high-fat diet resulted in an earlier onset of satiation.

Micrography performed on hypothalamic coronal sections showing the arcuate nucleus revealed no statistically significant differences in the total density of microglia among the experimental groups. However, activated microglial cells were more abundant in mice fed the high-fat diet compared to those on the standard diet. The cactus flour, in combination with the obesogenic diet, reduced the activated microglial density, albeit not reaching the same level observed in mice fed the standard diet. The authors concluded that *Opuntia ficus-indica* flour likely prevented obesity by mitigating the activation of microglial cells within the hypothalamic arcuate nucleus.

Using a cafeteria diet instead of a high-fat diet to induce obesity in rats, Chekkal et al. (2020) [22] sought to assess the impact of *Opuntia ficus-indica* cladodes on obesity and dyslipidemia in the rat model. Male Wistar rats were divided into two groups, with one receiving a cafeteria diet (CD group; 50% hyperlipidic diet + 50% junk food) either supplemented or not with *Opuntia ficus-indica* cladode extract (OFI group; 50 g/100 g diet), for 30 days. At the end of the experimental period, *Opuntia ficus-indica* extract prompted a reduction in both body weight (−20%) and adipose tissue weight (−45%). Moreover, a decrease in food intake (−10%) was also observed in rats fed the CD supplemented with *Opuntia ficus-indica* extract. Unfortunately, a pair-fed group was not included in the experimental design, preventing a clear distinction between the direct effects of the extract and those associated with the reduction in food intake. Regarding glycaemic control, a mitigation in serum glucose and insulin (−29 and −64%, respectively) levels was observed in the *Opuntia ficus-indica*-treated group. In addition, decreased glycated haemoglobin (−31%) and homeostasis model assessment of insulin resistance (HOMA-IR index −41%) were observed in this cohort in comparison with the control group. With regards to cholesterolaemia, the treated group showed a reduction in serum total cholesterol (−21%) level. Additionally, serum triglycerides and very-low-density lipoprotein-triglyceride (VLDL-triglycerides) levels decreased by 35% and 20%, respectively. In an effort to delve deeper into the mechanism of action underlying the effects induced by *Opuntia ficus-indica* extract, the authors focused on the oxidative status. Thus, they observed a significant decline in lipid peroxidation, as measured by thiobarbituric acid reactive substances (TBARS), in both the serum (−29%) and adipose tissue (−83%) of the *Opuntia ficus-indica*-treated group. This reduction in lipid peroxidation was also evident in VLDL. Furthermore, the authors assessed the enzymatic activity of paraoxonase 1 (PON-1), a protein involved in protecting against lipid peroxidation through the hydrolysis of xenobiotics. In fact, PON-1 plays an anti-atherogenic role, as this enzyme is bound to HDL and inhibits the oxidation of lipoproteins and lipids, decreasing the degree of inflammation [27]. In the *Opuntia ficus-indica*-treated group, there was a significant boost in serum PON-1 activity, showing a rise of 27% in serum and 47% in HDL-cholesterol. With regards to antioxidant enzymes, while superoxide dismutase (SOD) activity remained unchanged in serum, the activities of both glutathione peroxidase (GPx) and catalase (CAT) were elevated in the plasma of rats supplemented with *Opuntia ficus-indica* extract (+73% and +64%, respectively). In the adipose tissue, the *Opuntia ficus-indica*-treated group exhibited a significant rise in SOD and CAT activities (+67% and +40%, respectively). Collectively, these findings indicate that *Opuntia ficus-indica* cladodes contribute to preventing weight gain, improving glycemic balance and oxidative status.

Cladode extracts of *Opuntia ficus-indica* have also been investigated in alternative dietary models. In a study conducted by Cárdenas et al. (2019) [23], rats were subjected to a high-fructose diet, with *Opuntia ficus-indica* extract incorporated into their drinking water over a period of three weeks. Following the period, animals were maintained on the same diet and divided into three groups: (a) the control group, receiving only water, (b) the nopal group, where rats were orally administered 4.36 g/kg body weight per day of freshly daily prepared *Opuntia ficus-indica* cladodes extract, and (c) the mucilage group, wherein rats were orally administered 500 mg/kg body weight per day of mucilage fibre extract, over an additional 8-week period. The findings indicated that the rats receiving fructose in drinking water exhibited increased serum triglycerides and abdominal circumference. However, the administration of *Opuntia ficus-indica* cladodes extract significantly decreased plasma triglyceride levels (−43.4%, *p* < 0.05), with no observable changes in the abdominal circumference. In this study, the results suggested that the mucilage fibre extract was less effective than the *Opuntia ficus-indica* cladodes extract in achieving the observed effects.

In addition to cladode extracts, other *Opuntia ficus-indica* extracts have been used in several studies. Bounihi et al. (2017) [24] addressed a research to investigate the anti-adiposity and anti-inflammatory effects of prickly pear fruit vinegar of *Opuntia ficus-indica* in rats fed with an obesogenic diet for eight weeks. The control group received a standard diet, while an additional cohort was administered a high-fat diet (45% of energy from fat). Subsequently, three distinct groups were fed with the same high-fat diet supplemented with vinegar of the prickly pear at different doses: 3.5, 7 and 14 mL/kg body weight/day. The results revealed an elevation in both ultimate body weight and visceral adipose depot weights (mesenteric, epididymal and perirenal) among rats subjected to the high-fat diet. However, supplementation with prickly pear fruit vinegar demonstrated a significant mitigation of both body weight increase (−18.7%, −30.5% and −33% in final body weight by doses of 3.5, 7 or 14 mL/kg body weight/day, respectively) and total visceral fat depot increase (−18.7%, −30.5% and −33% in final body weight by doses of 3.5, 7 or 14 mL/kg body weight/day, respectively) across all administered doses. The high-fat feeding led to higher concentrations of plasma triglycerides, total cholesterol, low-density lipoprotein cholesterol (LDL-c) and coronary risk index (CRI). These effects were completely adverted through the administration of the prickly pear fruit vinegar, with efficacy demonstrated at all specified doses. The levels of leptin and TNF-α in both plasma and visceral adipose tissue were increased by the HFD, concomitant with a reduction in adiponectin levels. Prickly pear fruit vinegar successfully adverted these effects at all given doses.

Verón et al. (2019) [25] conducted an interesting study in an obese mice model which examined the beneficial effects of fermented *Opuntia ficus-indica* juice, either in isolation or fermented with a probiotic. For this purpose, *Opuntia ficus-indica* fruits, harvested in Argentina, were processed to obtain pasteurised juice. A portion of the juice was subsequently subjected to fermentation using *Lactobacillus plantarum* S-811, a strain previously isolated from *Opuntia ficus-indica*. The obese mice model was established by administering a high-fat diet to C57BL-6J mice. The animals were allocated into four experimental cohorts: the control group (C group) received a standard diet, the obese group was subjected to a high-fat diet (60.3% kcal as fat) (HFD group), a third group was fed with the high-fat diet supplemented solely with pasteurised *Opuntia ficus-indica* juice (5 mL/day/mouse), and a fourth cohort was administered the high-fat diet supplemented with pasteurised *Opuntia ficus-indica* juice and fermented with the probiotic (5 mL/day/mouse of 1.2 × 109 CFU/mL). This dietary regimen was maintained for seven weeks. In this study, the authors characterised the extract and observed a substantial enhancement in the content of both betanin and indicaxanthin (+41% and +38%, respectively) through the process of fermentation. These compounds are the main betalains present in this *Opuntia* species. Mice treated with the fermented *Opuntia ficus-indica* juice showed lower values in body weight, adipose tissue index, plasma triglycerides (−25%), total cholesterol (−25%), glucose (−25%), insulin (−35%) and HOMA-IR index (−51%), although these values did not reach the levels found in the control group. The non-fermented *Opuntia ficus-indica* juice failed to significantly mitigate either body weight or adiposity index in comparison with obese mice, although it did result in a notable reduction in both triglycerides and total cholesterol levels. Regarding leptin in plasma, obese mice showed an increase in leptin levels (hyperleptinemia), which were not prevented by the administration of *Opuntia ficus-indica* juices.

Due to the fact that probiotics, like *Lactobacillus plantarum* S-811, can regulate gut inflammation, the authors also determined interferon-γ (IFN-γ) and IL-10 cytokine levels. In this context, high-fat feeding did not increaseIL-10 levels in the serum, although it did significantly increase the concentration of gut IFN-γ. Nevertheless, within the gut, neither fermented nor non-fermented *Opuntia ficus-indica* juices induced alterations in these gut cytokines, in comparison with the obese mice group. Consequently, the authors concluded that fermented *Opuntia ficus-indica* juice exhibited anti-obesity properties, with the fermentation process enhancing the juice’s capacity to stimulate antioxidant defences.

The beneficial effects of *Opuntia* have also been analysed in combination with other foodstuffs. In the study reported by Rosas-Campos et al. (2022) [26], C57BL/6J mice were fed a high-fat diet (35% of energy from fat) in conjunction with high-carbohydrate beverage (2.31% fructose, 1.89% sucrose), for 16 weeks. In addition, during the initial eight weeks, the diet was supplemented with 10% *Opuntia ficus-indica* and 20% of a composite Mexican food mixture (referred to as MexMix), consisting of *Theobroma cacao* and *Acheta domesticus*, each comprising 10% *w*/*w*. As expected, the obesogenic diet caused an increase in mice body weight, visceral fat pad, epididymal fat pad, triglyceride levels, serum glucose and insulin levels, total- and LDL-cholesterol levels, glucose-dependent insulinotropic polypeptide (GIP), leptin, plasminogen activator inhibitor-1 (PAI-1) and resistin levels. Moreover, through hematoxylin-eosin staining, the authors observed that the high-fat diet increased adipocyte size and markers indicative of inflammatory infiltrates. Supplementation with MexMix significantly prevented all these effects (body weight −23%, visceral fat pad −71.7%, epididymal fat pad −39%, serum triglycerides −15.1%, serum glucose −22.5%, serum insulin −51%, total cholesterol −30.4%, LDL-cholesterol −73.3%, GIP -34.2%, leptin −74.4%, PAI-1 −41% and resistin −41%) and demonstrated the potential to reverse the reduction in insulin sensitivity induced by high-fat feeding. However, there were no changes in adiponectin, glucagon and serum adipokines. Taking into account that *Opuntia ficus-indica* was supplemented together with other foods, it became challenging to differentiate the specific impact of the cactus from the effects attributed to the other foods, or even the combined influence of all components.

As indicated previously, there is only one study documented in the literature that examines the effects of an *Opuntia* species different from *Opuntia ficus-indica*. Héliès-Toussaint et al. (2020) [18] not only investigated the effects of an eight-week supplementation with lyophilised cladode powder from *Opuntia ficus-indica* but also explored the impacts of cladode powder from *Opuntia streptacantha*. The study conducted an analysis of the proximal composition of the extracts, revealing statistical differences between both *Opuntia* species. *Opuntia streptacantha* presented a greater fibre content (6.52%), whereas *Opuntia ficus-indica* displayed the highest ash content (14.2%). Moreover, *Opuntia streptacantha* demonstrated the highest concentration of phenolic compounds and exhibited the highest antioxidant capacity. Specifically, in the case of *Opuntia streptacantha*, the concentration of gallic acid within phenolic acids was 65.1 µg/g, the quercetin content within flavonoids measured 19.0 µg/g, and the antioxidant activity was determined to be 897.8 µmol of Trolox/g sample. For *Opuntia ficus-indica*, the concentration of gallic acid within phenolic acids measured 56.7 µg/g, while the quercetin content in flavonoids was 20.4 µg/g. Additionally, the antioxidant activity was quantified as 659.4 µmol of Trolox/g sample.

In sharp contrast with *Opuntia ficus-indica*, the extract obtained from *Opuntia streptacantha* failed to prevent the final body mass gain. The supplementation reduced abdominal fat weight, serum glucose and insulin levels, as well as serum triglyceride levels, but these changes did not reach statistical significance. The decrease in serum adiponectin levels and the boost in leptin serum levels induced by the high-fat feeding were prevented by the *Opuntia streptacantha* extract. These results suggest that, in the assessment of the therapeutic potential of the two *Opuntia* species, the consumption of *Opuntia ficus-indica* appears to hold greater promise in the context of obesity and related metabolic alterations management.

### Summary

In summary, with the exception of a study specifically focused on a genetic obesity model (Zucker *fa*/*fa* rat), all the reported works analysing the effects of *Opuntia* products on obesity have been conducted in murine models, predominantly rats or mice, exhibiting diet-induced obesity. The induction methods for obesity in these experiments encompassed high-fat feeding, cafeteria diet or fructose supplementation. All the studies have centred on *Opuntia ficus-indica*, and the majority have analysed cladode extracts, although certain authors have employed alternative formulations such as fruit vinegar, fruit juice or flour. In addition, a single published study has been carried out using a fruit juice fermented with a probiotic, revealing this particular form to be more effective than its non-fermented counterpart. Taking into account that *Opuntia ficus-indica* products have been administered to animals concurrently with an obesogenic diet, the effects observed are indicative of their potential in preventing obesity.

**Figure 1:**
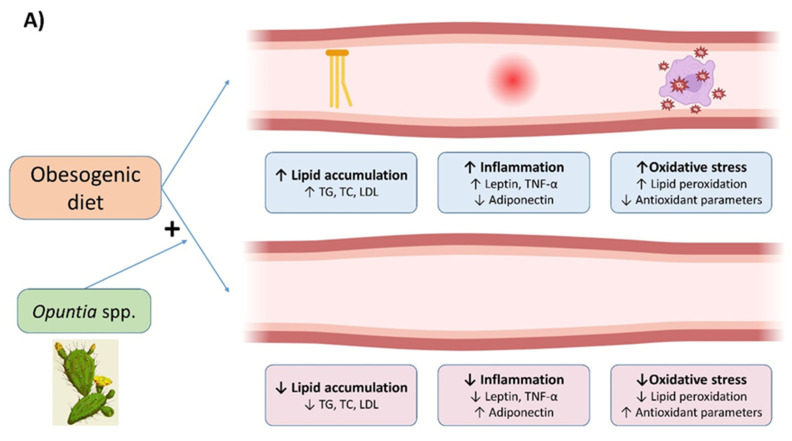

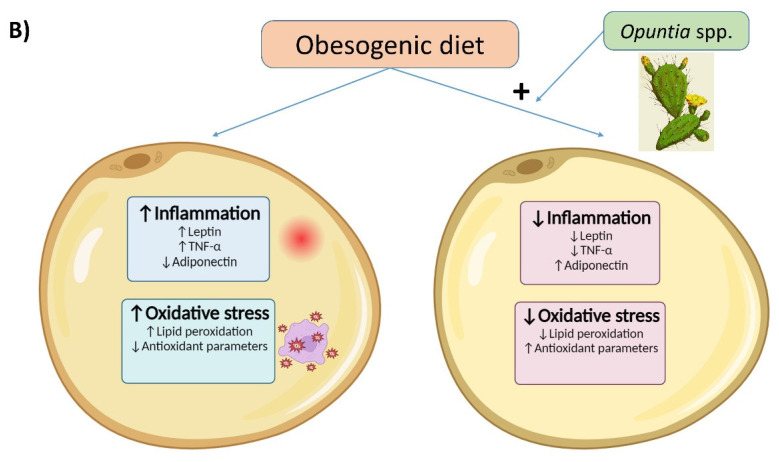
Graphical summary of the beneficial effects of *Opuntia* spp. in plasma (**A**) and adipose tissue (**B**) in animal models. TC: total cholesterol, TG: triglycerides, LDL: low-density lipoprotein, TNF-α: tumour necrosis factor-α; ↑: increase; ↓: decrease; +: diet supplementation.

The collective evidence from published studies consistently demonstrates that all *Opuntia ficus-indica* products employed possess a significant capacity to prevent, at least partially, obesity and certain associated co-morbidities, such as dyslipidemias and insulin resistance (Figure 1). These beneficial effects have been substantiated across different experimental period lengths, ranging from four to eight weeks. It is noteworthy that in the majority of the studies, the composition of bioactive compounds in *Opuntia ficus-indica* products has not been reported. Consequently, there is a lack of information concerning the main bioactive compounds present in these products that are responsible for the observed effects. This represents a significant limitation in the current understanding of the mechanisms underlying these benefits.

There is also a study that compares the effects of cladode extracts obtained from *Opuntia ficus-indica* and *Opuntia streptacantha*, with the former being more effective in managing obesity.

The potential mechanisms underlying the observed effects have been minimally explored, and the majority of reported works have not addressed this issue. Some authors have observed a reduction in markers of oxidative stress and inflammation, two key processes involved in the development of obesity. On the other hand, the improvement in insulin sensitivity induced by high-fat feeding may be related to changes in the serum adipokine profile. Alterations in microbiota composition could also be involved in the effects induced by *Opuntia ficus-indica*, although there is currently insufficient data to definitively support this assertion.

## 4. Effects of *Opuntia* spp. on Obesity and Related Co-Morbidities in Clinical Studies

In the context of studies involving human subjects, akin to those conducted in animal models, the predominant focus has been on analysing the effects of *Opuntia-ficus indica*. Notably, only one study has explored another *Opuntia* species (*Opuntia robusta*) (Table 2).

Linarès et al. (2007) [28] used the registered trademark NeOpuntia to study the effect of dehydrated leaves of *Opuntia-ficus indica* on metabolic syndrome. For this purpose, the authors carried out a randomised placebo-controlled trial involving 68 women (20–55 years-old) who exhibited metabolic syndrome and had a body mass index (BMI) ranging from 25 to 40 kg/m^2^. The participants were instructed to consume a supplement of *Opuntia* (1.6 g, three times daily; 35 women) or a placebo (33 women) for six weeks, alongside engaging in 30 min of daily physical activity. Within the placebo group, a significant reduction in HDL-C levels was observed. It is important to mention that, at the end of the study, 39% of the patients in the NeOpuntia group no longer met the diagnostic criteria for metabolic syndrome. Regarding the criterion of waist circumference > 80 cm, a component of metabolic syndrome, no alteration was noted in the placebo group after 42 days of treatment. However, within the intervention cohort (35 subjects), two volunteers no longer presented an increase in this parameter. Thus, the NeOpuntia supplement appears to contribute to the management of metabolic syndrome, although it is important to note that this study provides limited information on certain aspects.

Godard et al. (2010) [29] studied the effect of OpunDia™, a formulation comprising 75% *Opuntia ficus-indica* cladode extract and 25% fruit skin extract. The authors evaluated both acute and chronic beneficial effects in pre-diabetic obese men and women. To accomplish this objective, they conducted a double-blind, placebo-controlled study involving 29 volunteers (20 to 50 years old). Participants were randomly assigned to either the placebo group (14 subjects) or the intervention group (15 subjects), the latter receiving a daily dose of 400 mg of OpunDia™. In the chronic study, the experimental period length was sixteen weeks. In the acute treatment, a dosage of 400 mg of OpunDia™ was administered 30 min before the ingestion of a 75 g glucose solution. In this study, the authors ran an oral glucose tolerance test and observed a decrease in blood glucose concentrations at 60, 90 and 120 min following the administration of the *Opuntia* bolus. In the chronic study, no discernible effects of the *Opuntia* treatment were found between the two groups in terms of plasma insulin, proinsulin, high sensitivity C-reactive protein (hsCRP), adiponectin and glycated haemoglobin (HbA1c) levels. In addition, no changes were observed in fat mass, percent of body fat and total body weight during the chronic intervention. It is important to mention that OpunDia™ revealed no adverse effects on blood, liver or kidney parameters.

Grube et al. (2013) [30] conducted a randomised controlled trial to study the efficacy and safety of a natural fibre complex obtained from dehydrated cladodes of *Opuntia ficus-indica* (marketed under the commercial trademark Litramine IQP G-002AS). This complex was enriched with soluble fibre from *Acacia* spp. gum and coprocessed with cyclodextrin. It is noteworthy that this fibre complex, owing to its lipophilic activity, demonstrated the capacity to reduce the absorption of dietary fat by binding with fats in the intestinal tract, thereby forming fat–fibre complexes. The authors recruited obese and overweight subjects (*n* = 123 subjects, 30 male and 93 female) with a BMI ranging from 25 to 35 kg/m^2^ and aged between 18 and 60 years. Following a two-week placebo run-in phase, the study comprised a twelve-week treatment phase during which subjects received either 1000 mg of IQP G-002AS or a corresponding placebo, three times daily (after breakfast, lunch and dinner). Furthermore, all subjects were instructed to adhere to a mildly hypocaloric diet throughout the fourteen weeks of the study. The prescribed diet aimed to provide 55% of energy from carbohydrates, 30% from fat and 15% from protein, with a reduction in daily caloric intake by 500 kcal. In addition, subjects received instructions to increase their physical activity to 30 min/day. Under these experimental conditions, the fibre complex containing *Opuntia ficus-indica* induced a reduction in body weight (−2.4 kg) compared to the placebo group. Consequently, there was a decline in BMI, as well as in waist circumference (−1.7 cm) and body fat (−1.4 kg). The authors did not observe adverse effects after the ingestion of IQP G-002AS. Following the aforementioned study, the same authors in 2015 [31] conducted another double-blind, randomised study spanning twenty-four weeks. This design involved a cohort of subjects, both male and female volunteers with obesity aged between 18 and 60 years, with a BMI ranging from 25 to 35 kg/m^2^. Participants had also experienced a weight loss of at least 3% in the last 3–6 months. During the trial, the volunteers received 1 g of Litramine three times/day (intervention group; *n* = 25) or placebo (placebo group; *n* = 24). Concurrently, participants in both groups were advised to increase their physical activity, and no dietary restrictions were encouraged during the experiment. At the end of the experimental period, subjects in the Litramine group exhibited a significant decrease in body weight (−0.62 ± 1.55 kg) in comparison with the placebo group, where the mean body weight increased (+1.62 ± 1.48 kg). It is important to mention that subjects treated with Litramine showed lower BMI, waist and hip circumference than subjects from the placebo group. Regarding body fat content, the intervention cohort displayed a significant decrease in fat mass compared to the placebo group. Additionally, in terms of the feeling of satiety, 60% of the volunteers in the Litramine group reported experiencing satiety, while only 12.5% in the control group had a similar feeling

Pignotti et al. (2016) [32] conducted a study to evaluate the effects of *Opuntia ficus-indica* cladodes on improving oxidative stress and cardiometabolic risk factors in sixteen volunteers with moderated hypercholesterolaemia (LDL-C 120 mg/dL) and a BMI of 31.4 ± 5.7 kg/m^2^ at baseline. For this purpose, subjects were divided into two groups, with one group receiving *Opuntia* and the other serving as a control, with cucumber as their intake. Participants in the Nopal group adhered to their regular diet supplemented with one cup of boiled *Opuntia* cladodes twice a day (280 g/day), while the Cucumber group received one cup of boiled cucumber twice a day (260 g/day) as their dietary supplement. The intervention lasted two weeks following a washout period of 2–3 weeks. The results yielded no changes during the intervention period regarding BMI, body mass and percent body fat. On the other hand, both cucumber and nopal significantly increased plasma triglyceride levels, with no significant difference observed either in the response between the two groups or in changes in oxidative stress markers and lipoprotein subfractions. Therefore, based on the obtained results, the authors concluded that the potential of *Opuntia ficus-indica* cladodes to improve cardiometabolic risk and oxidative stress biomarkers cannot be asserted in patients with hypercholesterolaemia, at least within the experimental conditions of the study.

Aiello et al. (2018) [33] addressed an intervention study with 39 volunteers, aged 19–69 years, with at least two of these conditions: impaired glucose tolerance (fasting blood glucose ≥100 mg/dL), slight dyslipidemia (total cholesterol 190–240 mg/dL, triglycerides ≥150 mg/dL) or waist circumference ≥102 cm in men and ≥88 in women. The aim of the study was to analyse the antioxidant and anti-inflammatory properties of *Opuntia ficus-indica.* The intervention consisted of following a Mediterranean pattern diet along with consuming 500 g of pasta/week supplemented with 3% of cladode extract of *Opuntia ficus-indica* for 30 days, and comparing it with the group without nutritional intervention. After the experimental time, the results showed no significant differences in the percentage of fat mass or BMI in the intervention group compared to the control group; however, a significant reduction in abdominal waist was observed in both men (−2.3%) and women (−1.3%). The glycaemic levels of the intervention group were significantly reduced compared to the placebo group (−4%). Additionally, a trend towards reduced levels of serum total cholesterol was observed in the intervened group (−2.82%), although no significant changes were achieved. The authors concluded that their results confirmed the biological activity of *Opuntia* extracts, such as the hypoglycaemic effect observed, although more research is needed to determine a possible beneficial effect of extracts in the prevention of metabolic disorders.

In the same line, Giglio et al. (2020) [34] conducted a clinical study involving 49 Italian volunteers (13 male and 36 female), aged 40–65 years, with a mean BMI > 30 kg/m^2^. Subjects did not meet the requirements for a full diagnosis but exhibited one or two individual criteria associated with metabolic syndrome. Among all the participants, 31% presented hypertension, 12% were both obese and dyslipidemic, and 4% were diabetic. Subjects consumed 500 g of pasta supplemented with 3% *Opuntia ficus-indica* cladode extract (30% of insoluble polysaccharides) on a weekly basis over a period of one month. Participants followed the Mediterranean dietary pattern and engaged in limited physical activity. It is noteworthy that they did not alter the quantity of food consumed throughout the experimental period. After one month of treatment, BMI and body weight remained unchanged, while waist circumference (−1%), plasma glucose (−12.1%), triglycerides (−11.3%), creatinine (−2.7%) and aspartate transaminase (−16.3%) levels significantly decreased in comparison with baseline values. Moreover, although there was an increment in lesser amounts of atherogenic LDL-1, a reduction in denser LDL-2 (−26.2%) and LDL-3 (−44.6%) levels was observed.

Sánchez-Murillo et al. (2020) [35] addressed a study with 69 volunteer women (aged 40–60 years) having a BMI in the range of 27.5–29 kg/m^2^. Participants were divided into the control group (*n* = 13), without supplement administration, and the treated group (*n* = 56), supplemented with a daily dose of 5 g of *Opuntia ficus-indica* cladode in their morning meals over a period of twenty-four weeks. The *Opuntia ficus-indica* cladodes, harvested between 16 and 24 weeks of maturation, contained 52.75% carbohydrate, 13.51% protein, 1.55% fat and 5.83% crude fibre. No differences were found in body mass index or body fat.

Corona-Cervantes et al. (2022) [36] studied the potential of *Opuntia ficus-indica* to improve the health status of women with obesity by modifying the composition of intestinal microbiota. In this study, a sample of 36 female volunteers from Mexico (18–59 years) was included, with a BMI > 30 kg/m^2^ in the obesity group and falling within the range of 18.5–24.9 kg/m^2^ in the normal weight group. Participants were requested not to undergo antibiotic treatment in the three months prior to the study. The obesity group was subjected to an energy-restricted diet (−500 kcal/day) supplemented with 300 g of boiled *Opuntia ficus-indica*, obtained from 375 g of fresh cladodes. Additionally, participants were advised to engage in a 30 min daily walk for 30 days. The control group received an equivalent amount of *Opuntia ficus-indica* without any energy restriction or recommendation for walking. In the obesity cohort, the supplementation of *Opuntia ficus-indica* led to reductions in BMI (−2.8%), weight (−2.1%), hip (−0.6%), waist/hip ratio (−1%), serum levels of glucose (−12.5%), total cholesterol (−6.3%) and HDL cholesterol (−5.1%). However, no changes were observed in the normal weight group. It is important to mention that the average age differed significantly between cohorts, with 40.6 years in the obesity group and 22.1 years in the normal weight group. Furthermore, the authors studied the association between biochemical and anthropometric markers and the diversity of intestinal microbiota found in faecal samples, observing changes in the bacterial taxa in both cohorts. In the obesity group, there was an increasing tendency in *Prevotella, Roseburia, Lachnospiraceae* and *Clostridiaceae*, while *Bacteroides, Blautia* and *Ruminococcus* exhibited a decreasing trend. In fact, *Prevotella* species are associated with diets rich in fibre for their capacity to digest complex carbohydrates. In the normal weight group, following the intervention, a trend towards reduced amounts of *Ruminococcus* and *Bacteroides* and an increase in *Lachnospiraceae* family were observed. The decrease of *Bacteroidetes* could potentially be related to a higher intake of fructans obtained from *Opuntia*. The supplementation with *Opuntia ficus-indica* promoted the reduction in plasma total cholesterol, glucose and triglycerides. This effect could be attributed to the presence of polyphenols and soluble and insoluble fibres, which may influence the intestinal microbiota and, subsequently, cause alterations in the host metabolism. It is important to note that a limitation of the study is the difference in age between groups, given that age can impact the composition of microbiota.

Wolfram et al. (2002) [37] carried out the only reported study using a different *Opuntia* species. They analysed the effects of prickly pear pectin from *Opuntia robusta* on lipid and glucose metabolism. The investigation involved non-diabetic and non-obese males (37–55 years old), segmented into two groups: subjects with primary hypercholesterolemia (group A, *n* = 12) and those with combined hyperlipidaemia (group B, *n* = 12). The experimental period comprised an initial 8-week pre-running phase during which the volunteers followed a diet providing 7506 kJ (phase I). Subsequently, an additional eight weeks were included, wherein 625 kJ were replaced by prickly pear pulp (250 g/day) (phase II). The results revealed that the consumption of *Opuntia robusta* fruits induced a decrease in serum total cholesterol (−12%), LDL-cholesterol (−15%), apolipoprotein B (−9%), TG (−12%), fibrinogen (−11%), glucose (−11%), insulin (−11%) and uric acid (−10%). However, no changes were observed in body weight, cholesterol HDL, apolipoprotein A-I or lipoprotein levels. The hypocholesterolaemic action of *Opuntia robusta* may be partly explained by the presence of pectins in the *Opuntia* fruits.

### Summary

With the exception of one work, all the reported clinical trials conducted in humans have utilised *Opuntia ficus-indica*, the most extensively studied *Opuntia* species. The primary products employed in these studies include cladode extracts, dehydrated cacti, or derivatives such as flour or plant fibres. Additionally, one published article has investigated *Opuntia robusta*, with a specific focus on the use of prickly pear pulp.

Concerning the studies involving *Opuntia ficus-indica*, it is noteworthy that reductions in parameters related to obesity, such as body weight, BMI, waist circumference, hip circumference and fat mass, were observed only in certain instances, particularly those using a fibre extract or a cladode extract added to pasta. This observation underscores the significant influence of the experimental design on the outcomes. Furthermore, in several studies, certain prevalent obesity co-morbidities, such as hyperlipidemia and impaired glucose homeostasis control, have shown significant improvements. The beneficial effects on metabolic health do not consistently manifest in the same studies. Indeed, none of these works have conducted analyses to determine the mechanisms underlying the observed effects. In a particular study, the authors analysed the alterations induced by *Opuntia* supplementation in the composition of gut microbiota, but in fact, a definite causality with changes in other observed parameters has not been firmly established.

Regarding *Opuntia robusta*, it appears that the fruit pulp may be beneficial in managing some serum lipid alterations associated with obesity, although its efficacy in reducing body fat remains inconclusive. Nevertheless, only one study has been conducted with this *Opuntia* species, making it challenging to draw definite conclusions.

## 5. Conclusions

The data documented in the literature and compiled in the present review provide scientific evidence supporting the effect of *Opuntia ficus-indica* on obesity and some related metabolic disorders. Considering that published studies conducted in animal models have explored the effects of this plant in animals subjected to an obesogenic feeding pattern, wherein *Opuntia ficus-indica* products were administered concurrently with the obesogenic diet, the conclusion that can be drawn from these studies is that *Opuntia ficus-indica* has the potential to prevent obesity. Consequently, additional studies are warranted to assess its efficacy in the treatment of obesity. Nevertheless, there is a notable scarcity or lack of knowledge concerning crucial aspects. Thus, very limited information has been provided regarding the mechanisms of action underlying the observed effects. Conversely, the absence of a characterisation of the bioactive compound profile of *Opuntia ficus-indica* products means that information concerning the main molecules responsible for the observed effects is not available. Hence, it is crucial to undertake chemical characterisation to standardise the extracts that demonstrate beneficial effects on health. Moreover, it is important to precisely state which kind of product, whether cladode extract, fruit extract, fruit juice, etc., represents the optimal choice.

The effects observed in humans are not as straightforward as those found in animal models, with this discrepancy potentially arising from several reasons, including the inter-individual variability in humans compared to animals. In this line, in animal studies, all subjects within each experimental group typically exhibit similar characteristics, a circumstance that may not always be replicated in human subjects. For instance, in some studies, participants may be either overweight or obese, and the possibility of a differential response between these groups cannot be discarded. Moreover, when comparing various studies, the metabolic characteristics of the participants can vary significantly; thus, in some studies, volunteers may exhibit metabolic syndrome, while in others, they may not. In addition, a wide variety of *Opuntia* products has been used. As a result, further studies should be conducted across diverse population groups to enhance generalisability and understanding.

In summary, based on the existing scientific evidence, *Opuntia*, particularly *Opuntia ficus-indica*, emerges as a promising botanical resource for the development of products containing bioactive compounds that could prove beneficial in the management of obesity and its co-morbidities. Nevertheless, the existing knowledge is still limited, and further research is needed in both animal models and humans to conclusively determine whether it truly represents an effective tool for use.

## Figures and Tables

**Table 1:** Effects of *Opuntia* products in animal models.

Author Year [Ref.]	Animal Model	*Opuntia* Species and Product	Experimental Design	Effects	Mechanism
Morán-Ramos et al., 2012 [10]	Male Zucker (*fa*/*fa*) rats (7-week-old)	*Opuntia ficus-indica* Dehydrated extract of cladodes	Standard diet supplemented or not with 4% of dietary fibre from *Opuntia ficus-indica* in place of cellulose 7 weeks	NS Final body weight ↑ Food intake ↓ Cholesterol, ALT, AST serum levels ↑ Adiponectin serum level	
Aboura et al., 2017 [19]	Swiss male mice (40–50 g weight)	*Opuntia ficus-indica* Aqueus cladode extract	High-fat diet (60% of energy from fat) or standard diet supplemented or not with 1% of extract in the drinking water 6 weeks	↓ Body weight ↓ Adipose tissue weight ↓ TG, TC, glucose, insulin, IL-6 and TNFα plasma levels ↓ *Leptin, Il-1β, Il-6* and *Tnfα* gene expression in adipose tissue	
Sánchez-Tapia et al., 2017 [20]	Male Wistar rats (6 week-old)	*Opuntis ficus-indica* Dehydrated extract of cladodes to provide 5% of dietary fibre from nopal in place of cellulose	High-fat diet (45% of energy from fat added to the diet and 5% sucrose added to the drinking water) or standard diet 7 months Each group continued with the same diet but were distributed into 4 groups: rats fed the standard diet or the high-fat diet supplemented or not with nopal 1 month	↓ Body weight (SD+*Opuntia*; HFD+*Opuntia*) ↓ Glucose and insulin serum levels (SD+*Opuntia*; HFD+*Opuntia*) ↓ TG, TC and leptin serum levels (SD+*Opuntia*; HFD+*Opuntia*) ↓ LDL-cholesterol serum levels (SD+*Opuntia*) ↓ *Leptin, Nox* and *App* gene expression in adipose tissue (SD+*Opuntia*; HFD+*Opuntia*) ↓ *Tnf*α gene expression in adipose tissue (HFD+*Opuntia*)	↑ Gut microbiota diversity and abundance ↑ *Bacteroidetes* increased with respect to the *Firmicutes* ↑ *Anaeroplasma, Prevotella* and *Ruminucoccus* (SD+*Opuntia*; HFD+*Opuntia*) ↓*Faecalibacterium, Clostridium* and *Butyricicoccus* (SD+*Opuntia*; HFD+*Opuntia*) ↑ Intestinal mucus layer thickness and occluding (HFD+*Opuntia*) ↓ LPS serum levels (HFD+*Opuntia*)
Héliès-Toussaint et al., 2020 [18]	Male Sprague–Dawley rats (6 week-old)	*Opuntia ficus-indica* (OFI) *Opuntia streptacantha* (OSC) and Cladode extracts (0.5% *w*/*w* in the diet)	HFD (30% of energy from fat) 8 weeks	↓ Body mass gain (significant only in OFI) ↓ Serum adiponectin level (both OSC and OFI) ↑ Serum leptin level (both OSC and OFI)	↑ Faecal lipid excretion (OFI)
Urquiza-Martínez et al., 2020 [21]	Male C57Bl/6J mice (9 week- old)	*Opuntia ficus-indica* Flour (17% *w*/*w* in the diet)	HFD (60% of energy from fat) or SD 12 weeks 4 additional weeks with supplementation or not 17% cactus	↓ Body weight ↓ Epididymal and retroperitoneal adipose tissue weights ↓ AUC in glucose and insulin tolerance tests	↑ Caloric intake (SD+*Opuntia*) ↓ Caloric intake (HFD+*Opuntia*) Delays the point of satiation (SD+*Opuntia*) Earlier point of satiation (HFD+*Opuntia*) ↓ Activated microglial cells in arcuate nucleus (HFD+*Opuntia*)
Chekkal et al., 2020 [22]	Male Wistar rat (4 week-old and 110 ± 20 g)	*Opuntia ficus-indica* Cladode extract (50 g/100 g diet)	Cafeteria diet (50% hyperlipidic diet with 20% of energy from fat + 50% junk food) 4 weeks	↓ Body weight ↓ Adipose tissue weight ↓ Food intake ↓ Serum glucose, insulin and glycated haemoglobin levels and HOMA-IR ↓ Serum TC, TG, TG-VLDL levels	↓ Lipid peroxidation in serum ↑ PON-1, GPx and CAT activities in serum ↓ Lipid peroxidation in adipose tissue and VLDL ↑ PON-1 activity in HDL ↑ SOD and CAT activities in adipose tissue
Cárdenas et al., 2019 [23]	Male Wistar rats (8 week-old and 250–350 g)	*Opuntia ficus-indica* Cladode extracts (4.36 g/kg bw/day)	Standard diet + 20% fructose in water 3 weeks + additional 8 weeks, receiving or not cactus extract	↓ Plasma TG levels NS Abdominal circumference	
Bounihi, A. et al., 2017 [24]	Male Wistar rats (7–8 week-old)	*Opuntia ficus-indica* Vinegar of prickly pear (fruit)	High-fat diet (45% of energy from fat) supplemented with 3.5, 7 or 14 mL/kg/day of the vinegar 8 weeks	↓ Final body weight (all doses) ↓ Visceral adipose tissue weights (mesenteric, epididymal and perirenal) (all doses) ↓ TG, TC, LDL-c and CRI plasma levels (all doses) ↑ Adiponectin in plasma (all doses) ↓ Leptin and TNF-α in plasma (all doses)	
Verón et al., 2019 [25]	Male C57BL-6 mice (4–6 week-old)	*Opuntia ficus-indica* Fruit juice (5 mL/day/mouse) fermented and non- fermented with *Lactobacillus plantarum* S-811(1.2 × 10^9^ CFU/mL)	High-fat diet (60.3% of energy from fat) 7 weeks	↓ Body weight ↓ Adipose tissue index (HFD-fermented OFI group) Fermented fruit juice: ↓ Plasma TG, TC, glucose and insulin levels and HOMA-IR index (HFD-fermented OFI group) Non-fermented fruit juice: ↓ Plasma TG and TC levels NS Blood leptin levels	
Rosas-Campos et al., 2022 [26]	Male C57BL/6J mice (7 week-old)	*Opuntia ficus* *Indica* along with other 2 Mexican functional foods (MexMix): *Theobroma cacao* and *Acheta domesticus* (10% from 8th to 16th each)	HFD (35% of energy from fat) together with high-carbohydrate beverage (2.31% fructose, 1.89% sucrose) From week 8th to week 16th supplementation	↓ Body weight ↓ Visceral and epididymal fat pad ↓ Adipocyte size (hyperplasia) ↓ Variations in adipocyte size and shape ↓ Serum glucose and insulin levels ↑ Insulin sensitivity ↓ Serum TG, cholesterol and LDL cholesterol levels ↓ Inflammatory infiltrates (by haematoxylin-eosin staining)	

ALT: alanine transaminase; APP: amyloid precursor protein; AST: aspartate transaminase; AUC: area under the curve; CAT: catalase; CFU: colony forming unit; CRI: coronary risk index; HFD: high-fat diet; HOMA-IR: homeostatic model assessment for insulin resistance; IL: interleukin; LDL: low-density lipoprotein; LPS: lipopolysaccharide; NOX: NADPH oxidase; NS: Not significant; PAI-1: plasminogen activator inhibitor-1; PON: paraoxonase; SD: standard diet; SOD: superoxide dismutase; TC: total-cholesterol; TG: triglycerides; TNFα: tumour necrosis factor α; VLDL: very-low-density lipoprotein-triglycerides; ↑: increase; ↓: decrease.

**Table 2:** Clinical studies addressed with *Opuntia* products.

Author Year [Ref.]	Participants	*Opuntia* Species, Product and Dose	Experimental Design	Effects	Mechanism
Linarès et al., 2007 [28]	59 women with metabolic syndrome Age: 20–55 year-old BMI: 25–40 kg/m^2^	*Opuntia ficus-indica* Dehydrated leaves (NeOpuntia) 1.6 g, 3 times daily	Intervention group and placebo group 6 weeks All subjects: 30 min physical activity/day	↑ HDL-cholesterol ↓ Triglycerides ↓ Waist circumference ↓ Patients with metabolic syndrome (−39%)	
Godard et al., 2010 [29]	29 pre-diabetic adult male and female (14 placebo and 15 patients Age: 20–50 year-old BMI: 30–35 kg/m^2^	*Opuntia ficus-indica* OpunDia™ (a capsule contains 75% *Opuntia ficus-indica* cladode extract + 25% fruit skin extract) 400 mg/day	Intervention group and placebo group Acute study: OpunDia^TM^ were given 30 min before ingestion of a 75 g glucose solution Chronic study: 16 weeks	Acute study phase: ↓ Plasma glucose level at 60, 90 and 120 min Chronic study phase: NS fat mass, percent body fat and total body weight NS Plasma insulin, proinsulin, hsCRP, adiponectin and HbA1c levels	
Grube et al., 2013 [30]	123 volunteers (30 male and 93 female) Age: 18–60 year-old BMI: 25–35 kg/m^2^	*Opuntia ficus-indica* Litramine IQP G-002AS (a fibre extract) 3000 mg/day	Intervention group and placebo group All subjects: hypocaloric diet (−500 kcal/day) plus daily exercise (30 min/day) 14 weeks	↓ Body weight ↓ BMI ↓ Waist circumference ↓ Body fat	
Grube et al., 2015 [31]	49 volunteers Age: 18–60 year-old BMI: 25–35 kg/m^2^	*Opuntia ficus-indica.* Litramine IQP G-002AS (a fibre extract) 3000 mg/day	Intervention group (*n* = 25): received 1000 mg fibre extract three times a day Placebo group (*n* = 24): received 1000 mg of cellulose three times a day All subjects: indication of daily exercise (30 min/day) 24 weeks	↓ Body weight ↓ BMI ↓ Hip and waist circumference ↓ Fat mass ↓ Satiety	
Pignotti et al., 2016 [32]	16 participants with moderate hypercholesterolemia (LDL-c ≥ 120 mg/dL) Age: 32–60 year-old BMI: 31.4 ± 5.7 kg/m^2^	*Opuntia ficus-indica* Pad boiled 280 g/day	Nopal group: received 1 cup (140 g) of *Opuntia* twice a day Cucumber group (control): received 1 cup (130 g) of cucumber twice a day 2 weeks	NS BMI, body mass and% fat ↑ Plasma triglycerides	
Aiello et al., 2018 [33]	39 participants with at least two of these conditions: impaired glucose tolerance, slight dyslipidaemia or waist circumference ≥102 cm in men and ≥88 in women Age: 19–69 year-old	*Opuntia ficus-indica* 500 g of pasta/week supplemented with 3% of cladode extract	Intervention group and placebo group All subjects: Mediterranean diet 4 weeks	NS% fat mass and BMI ↓ Abdominal waist circumference in men and women ↓ serum glycemia NS serum total cholesterol	
Giglio et al., 2020 [34]	49 volunteers (13 male and 36 female without metabolic syndrome. Among the 49 subjects: 31% presented hypertension, 12% were obese and dyslipidaemic, and 4% were diabetic Age: 40–65 year-old BMI: > 30 kg/m^2^	*Opuntia ficus-indica* 500 g of pasta/week supplemented with 3% of cladode extract (30% of insoluble polysaccharides)	Intervention group and placebo group All subjects: Mediterranean diet and little physical activity was practiced 4 weeks	NS Body weight and BMI ↓ Waist circumference ↓ Plasma glucose, triglycerides, creatinine and AST ↓ LDL-2 and LDL-3	
Sánchez-Murillo et al., 2020 [35]	69 women volunteers Age: 40–60 year-old BMI: 27.8 and 29.0 kg/m^2^	*Opuntia ficus-indica* Flour from cladodes 5 g/day	Intervention group (*n* = 56); and control group (*n* = 13) 24 weeks	NS BMI and body fat	
Corona-Cervantes et al., 2022 [36]	36 women Age: average age between groups were significantly different (obesity group: 40.6 year-old and normal weight group: 22.1 year-old) BMI: >30 kg/m^2^ in obesity group; BMI: 18.5–24.9 kg/m^2^ in normal weight group	*Opuntia ficus-indica* Boiled fresh cladodes 300 g/day	Intervention group (obesity group); and control group (normal weight group) No antibiotic treatment in the three months prior to the study	Obesity group: ↓ BMI, weight, hip, waist/hip ratio, serum glucose, total cholesterol and HDL-cholesterol Normal weight group: no changes	Obesity group ↑ *Prevotella, Roseburia, Lachnospiraceae* and *Clostridiaceae* ↓ *Bacteroides, Blautia* and *Ruminococcus* Normal weight group: ↓ *Ruminococcus* and *Bacteroides* ↑ *Lachnospiraceae*
Wolfram et al., 2002 [37]	24 non-diabetic, non-obese males with hypercholesterolemia or hyperlipidaemia Age: 37–55 year-old	*Opuntia robusta* Prickly pear pulp 250 g/day	Group A: Patients with primary isolated hypercholesterolemia (*n* = 12) Group B: Patients with combined hyperlipidaemia (*n* = 12) 16 weeks Phase I: 8 weeks of pre-running phase with a diet of 7506 kJ Phase II: 8 weeks with a diet where 625 kJ were replaced by prickly pear pulp	NS Body weight ↓ Plasma total cholesterol, LDL-cholesterol, apolipoprotein B, triglycerides, fibrinogen, glucose, insulin and uric acid NS HDL-cholesterol, apolipoprotein A-I, and lipoprotein(a)	

AST: aspartate aminotransferase; BMI: body mass index; HbA1c: glycated haemoglobin; HDL: high-density lipoprotein; hsCRP: High sensitivity C-reactive protein; LDL: low-density lipoprotein; NS: not significant; ↑: increase; ↓: decrease.

## Data Availability

The authors confirm that the data supporting the findings of this study are available within the article.
